# Medicinal plants and natural products in amelioration of arsenic toxicity: a short review

**DOI:** 10.1080/13880209.2016.1235207

**Published:** 2016-12-08

**Authors:** Sanjib Bhattacharya

**Affiliations:** West Bengal Medical Services Corporation Ltd, Salt Lake City, Kolkata, West Bengal, India

**Keywords:** Arsenicosis, ascorbic acid, antioxidant, curcumin, clinical

## Abstract

**Context:** Chronic arsenic toxicity (arsenicosis) is considered a serious public health menace worldwide, as there is no specific, safe, and efficacious therapeutic management of arsenicosis.

**Objectives:** To collate the studies on medicinal plants and natural products with arsenic toxicity ameliorative effect, active pre-clinically and/or clinically.

**Methods:** Literature survey was carried out by using Google, Scholar Google and Pub-Med. Only the scientific journal articles found on the internet for last two decades were considered. Minerals and semi-synthetic or synthetic analogs of natural products were excluded.

**Results:** Literature study revealed that 34 medicinal plants and 14 natural products exhibited significant protection from arsenic toxicity, mostly in preclinical trials and a few in clinical studies.

**Conclusion:** This research could lead to development of a potentially useful agent in clinical management of arsenicosis in humans.

## Introduction

Arsenic is the 20th most abundant natural element ubiquitous in earth’s crust and biosphere. It is introduced into soil and groundwater during weathering of rocks followed by subsequent leaching and runoff. It can also be introduced into soil and ground water from anthropogenic activities (Singh 2006). Humans are exposed to arsenic predominantly through contaminated drinking water, whereas inhalation and skin absorption are minor routes of exposure (Shi et al. 2004). Chronic arsenic exposure through drinking water to humans leads to carcinogenesis of almost all organs, skin diseases (viz. hyper-pigmentation, hyperkeratosis) leading to cancers of skin and epithelial tissues; hepatic, renal, cardiovascular, respiratory, central nervous system, gastrointestinal, reproductive complications and children’s intellectual impairment; thereby increasing morbidity and mortality (Kapaj et al. 2006; Mazumder 2008). Chronic arsenic toxicity (arsenicosis) due to drinking of arsenic contaminated ground water is a major environmental public health hazard throughout the world especially affecting India and Bangladesh.

Arsenicosis leads to irreversible damage in several vital organs and arsenic is an established carcinogen. Despite the magnitude of this potentially fatal toxicity, there is no effective therapy for this disease; patients once affected may not recover even after remediation of the arsenic contaminated water. Arsenic toxicity is considered as a serious problem worldwide, as there is no specific, safe and efficacious therapeutic management of arsenicosis. The need for an effective therapy for arsenicosis is therefore obvious (Ratnaike 2003; Mazumder 2008).

Chelation therapy for arsenic toxicity is thought to be the specific therapy for relief of systemic clinical manifestations and reduction of arsenic stores in the body, reducing subsequent cancer risk. Chelating agents, namely DMSA (dimercaptosuccinic acid), DMPS (dimercaptopropane succinate) and d-penicillamine were previously tried for treatment of chronic arsenic toxicity. However, their clinical usefulness for management of arsenicosis is yet to be established (Mazumder et al. 1998, 2001; Sun et al. 2006). No treatment of proven benefit is currently available for routine use for arsenocosis patients. Treatment options advocated are vitamin and mineral supplements and antioxidant therapy (Ratnaike 2003).

The toxic effects of arsenic in human body and their conventional managements so far have been well studied and reviewed earlier (Abdul et al. 2015). But there is no comprehensive account on the studies on the alternative options for counteracting of arsenic toxicity.

The use of plant and plant products for treatment of diseases is as old as mankind. The major merits of plant based medicine seem to be their perceived efficacy, low incidences of serious adverse effects and low cost (Bhattacharya & Haldar 2013a). Literature survey reveals that from the last 10 years only experimental research has been escalated in pursuit of medicinal plants and natural products (phytochemicals) that could abrogate arsenic toxicity in animals and humans. Several medicinal plants and phytochemicals exhibited significant protection form experimentally induced arsenic toxicity in animal models. The objective of the present review is to summarize relevant preclinical and clinical research findings in this area.

## Methods

Internet-assisted literature study was carried out by using Google, Scholar Google, and Pub-Med database search. Only the scientific journal articles published and/or abstracted in internet during last two decades (1996–2016) were considered. The experimental preclinical and clinical studies on medicinal plants (crude, semi-pure, or enriched extracts thereof) and natural products were selected. Combination of natural products was regarded as a separate study. Here, minerals were not contemplated as natural products. The semi-synthetic or synthetic analogues of natural products were excluded from the present scope of compilation.

## Results

### Preclinical studies

#### Medicinal plants

Thirty-four medicinal plants are reported to possess arsenic toxicity ameliorative property in sub-chronic arsenic toxicity in experimental models. The details are summarized in Table 1. Most of the studied plants are indigenous to the Indian subcontinent. These include certain putative medicinal plants recognized in Ayurveda, the traditional system of Indian medicine and worldwide, namely, *Withania somnifera, Mentha piperita, Emblica officinalis (Phyllanthus emblica), Azadirachta indica, Boerhavia diffusa, Camellia sinensis, Vitis vinifera, Terminalia arjuna, Moringa oleifera, Ocimum sanctum* and *Allium sativum.* The edible plants include *Camellia sinensis, Vitis vinifera*, *Zea mays, Triticum aestivum, Trichosanthes dioica*, *Carica papaya*, *Spinacia oleracea* and *Allium sativum.* The less known plants showing such effects in multiple organ systems of rodents include *Ipomea aquatica, Trichosanthes dioica* and *Corchorus olitorius.* Lower plant (algae) possessing this property is *Spirulina*.

**Table 1.** Medicinal plants with arsenic toxicity ameliorative potential.

Sl. No.	Botanical name	Part/Constituents used	Experimental Model	Organ(s)/System/Cell line incolved	References
1	*Withania somnifera*	Root	Rats	Testes, liver, kidney	Kumar et al., 2015a,b
2	*Ipomea aquatica*	Aerial parts	Mice	Liver, kidney, heart, brain and testes	Dua et al., 2015
3	*Mentha piperita*	Leaf	Mice	Liver	Sharma et al., 2007
4	*Carica papaya*	Fruit	Mice	Testes	Singh & Kumari 2013
5	*Phyllanthus emblica*	Leaf	Mice	Liver, kidney and spleen	Sayed et al., 2015
6	*Emblica officinalis*	Fruit	Mice	Thymocytes	Singh et al., 2013
7	*Pteris longifolia*	Leaf	Rats	Liver, kidney	Kumar et al., 2015b
8	*Triticum aestivum*	Leaf	Rats	Liver, kidney	Lakshmi et al., 2015
9	*Azadirachta indica*	Leaf	Rats	Liver	Oyewole 2011
10	*Tephrosia purpurea*	Aerial parts	Rats	Liver	Baxla et al., 2014
11	*Irvingia gabonensis*	Leaf	Rats	Liver	Gbadegesin et al., 2014
12	*Eupatorium buniifolium*	Aerial parts	–	Renal Vero cells	Soria et al., 2008
13	*Lantana grisebachii*	Aerial parts	–	Renal Vero cells	Soria et al., 2008
14	*Mandevilla pentlandiana*	Aerial parts	–	Renal Vero cells	Soria et al., 2008
15	*Sebastiania commersoniana*	Aerial parts	–	Renal Vero cells	Soria et al., 2008
16	*Heterothalamus alienus*	Aerial parts	–	Renal Vero cells	Soria et al., 2008
17	*Boerhavia diffusa*	Aerial parts	–	H9c2 cardiomyocytes	Vineetha et al., 2013
18	*Camellia sinensis*	Black and green tea	Mice	Liver	Sinha et al., 2010
19	*Camellia sinensis*	Green tea	Rats	Liver	Acharyya et al., 2014
20	*Camellia sinensis*	Black and green tea	Rabbits	Haematological	Raihan et al., 2009
21	*Camellia sinensis*	Tannin-rich fraction of green tea	Rats	Liver and Kidney	Chandronitha et al., 2010
22	*Malus domestica*	Peel	–	H9c2 cardiac myoblast cells	Vineetha et al., 2014
23	*Vitis vinifera*	Seed proanthocyanidin extract	Mice	Testes	Li et al., 2015
24	*Vitis vinifera*	Seed	Rats	Liver	Xinjuan et al., 2011
25	*Lantana grisebachii*	Phyto-extract	–	Lymphocyte cells	Soria et al., 2014
26	*Chlorophytum borivilianum*	Root	Mice	Testes	Sharma & Kumar 2014
27	*Terminalia arjuna*	Whole plant	–	Chicken liver cell	Verma et al., 2007
28	*Phyllanthus fraternus*	Whole plant	–	Chicken liver cell	Verma et al., 2007
29	*Trichosanthes dioica*	Root	Rats	Liver, kidney, heart, brain	Bhattacharya & Haldar 2012a,b; 2013b
30	*Trichosanthes dioica*	Fruit	Rats	Liver, kidney, heart	Bhattacharya & Haldar 2012c, Bhattacharya et al., 2014
31	*Moringa oleifera*	Whole plant	–	Chicken liver cell	Verma et al., 2007
32	*Moringa oleifera*	Seed	Rats	Liver, Kidney	Gupta et al., 2005
33	*Moringa oleifera*	Leaf	Mice	Heart, Liver, Kidney	Sheikh et al., 2014
34	*Corchorus olitorius*	Leaves	Rats	Brain, Liver, Kidney, Heart	Das et al., 2010a, b, c
35	*Psidium guajava*	Leaves	Rats	Kidney, Haematological	Roy & Roy 2011; Tandan et al., 2012
36	*Ocimum sanctum*	Leaves	Rats	Liver, Kidney	Banu et al., 2009
37	*Allium sativum*	Bulb	Mice	Bone marrow	RoyChoudhury et al., 1996
38	*Allium sativum*	Bulb	Rats	Liver, Kidney, Ovary, Erythrocytes	Chowdhury et al., 2008; Adegboyega & Odunola 2012
39	*Allium sativum*	Bulb	–	Human malignant melanoma cells (A375), human keratinocyte cells (HaCaT), human normal dermal fibroblast cells	Chowdhury et al., 2008
40	*Viscum album*	Leaf	Rats	Erythrocytes	Adegboyega & Odunola 2012
41	*Eichhornia crassipes*	Root	Rats	Liver, spleen, kidney, lungs, skin	Quayum 2007
42	*Zea mays*	Fruit	Rats	Liver, kidney, heart, lungs, skin	Chowdhury et al., 2009
43	*Spinacia oleracea*	Aerial parts	Rats	Liver, spleen, kidney, lungs, skin	Umar 2007
44	*Spirulina*	Whole plant (algae)	Rats	Liver cells	Saha et al., 2005

Except garlic (juice) in most of the cases the crude extracts of dried plant materials using suitable solvents are used for the studies. In case of *Camellia sinensis* (tea leaf), and *Vitis vinifera* (grape seed) specific chemical constituent enriched extracts were employed and found beneficial effects in ameliorating multiple organ toxicities in rodents.

Except cells/cell lines most common intact models include rodents like mice and rats. Most commonly studied parameters are hematological and antioxidative parameters. Parameters specific for organs include those of liver, kidney, heart, brain, testes; while liver being the most common. Histopathology of these organs was also performed in some cases. Measurement of arsenic contents in concerned tissues was performed in few cases. Sodium arsenite (NaAsO_2_) is used most commonly as toxicant.

#### Natural products

Fourteen natural products were found to demonstrate arsenic-induced sub-chronic toxicity ameliorative effects mostly in intact rodent models. The details are summarized in Table 2. Among them three are vitamins, namely, ascorbic acid (vitamin C), α-tocopherol (vitamin E) and all-trans retinoic acid (vitamin A acid). Except the last one, rests are phytochemicals. Ascorbic acid, α-tocopherol and quercetin are also used as reference compounds in above mentioned studies on medicinal plant extracts. Ascorbic acid and α-tocopherol co-administration showed prominent ameliorative effect in several animal studies by modulating oxidative stress and apoptosis; indicating prospect of this combination for clinical regimen.

**Table 2.** Natural products with arsenic toxicity ameliorative potential.

Sl. No.	Name	Experimental model	Organ(s)/System/Cell line involved	References
1	Rutin	Rats	Behavioural and general	Sárközi et al., 2015
2	β-Carotene	Mice	Liver, Kidney	Das et al., 2015
3	Leutin	Mice	Testes, liver	Niu et al., 2015; Li et al., (2016)
4	Diallyl trisulfide	Rats	Erythrocytes and lymphocytes	Prabu & Sumedha 2014
5	Silibinin	Rats	Kidney, Liver	Prabu & Muthumani 2012; Muthumani & Prabu 2012; 2013
6	Naringenin	Mice	Liver, Kidney	Roy et al., 2014
7	Naringenin	Rats	Liver, Kidney	Mershiba et al., 2013
8	Genistein	Rats	Heart	Fan et al., 2013
9	Ascorbic acid	Rats	Liver, Kidney, Haematogical	Singh & Rana 2007; Rana et al., 2010
10	Ascorbic acid	Mice	testes	Chang et al., 2007
11	α-Tocopherol	Mice	Liver, Kidney	Verma et al., 2004; Mittal & Flora 2007
12	Ascorbic acid + α-Tocopherol	Rats	Testes, Brain	Mukhopadhyay et al., 2013; Herrera et al., 2013; Kadirvel et al., 2007; Ramanathan et al., 2002, 2005.
13	Curcumin	Mice	Liver	Biswas et al., 2010; Gao et al., 2013
14	Curcumin	Rats	Liver, brain	Sankar et al., 2015; Yousef et al., 2008
15	Curcumin	–	Human Lymphocytes	Mukherjee et al., 2007
16	Quercetin	Rats	Liver, brain, testes	Ghosh et al., 2009; Jahan et al., 2015
17	Resveratrol	Cats	Liver, brain, lung	Zhang et al., 2014; Cheng et al., 2013, 2014
18	Resveratrol	Rats	Lung, Liver	Zhang et al., 2013a, b
19	Resveratrol	Mice	Heart	Zhao et al., 2008
20	All-*trans* retinoic acid	Rats	Uterus	Chatterjee & Chatterji 2011
21	Arjunolic acid	Mice	Liver, heart, brain, kidney, testes	Manna et al., 2007, 2008a, b; Sinha et al., 2008a, b

Development of novel formulation or pharmaceutical delivery systems like liposome and nanoencapsulation in case of quercetin, nanoencapsulation for curcumin improves the efficacy than their conventional administration in rodents. Commonly studied parameters were hematological and antioxidative parameters for organs as stated above. Histopathology of these organs was also performed in some cases. Measurement of arsenic contents in concerned tissues was performed in a few cases. Sodium arsenite (NaAsO_2_) and arsenic trioxide (As_2_O_3_) were both used as toxicant.

### Clinical studies

All of these studies were carried out in Bangladesh. The clinically active agents are listed in Table 3. The putative dietary supplement *Spirulina* alone and in combination with zinc were found to be beneficial in patients of chronic arsenic poisoning (Sikder et al. 2000; Khan et al. 2001; Misbahuddin et al. 2006). Oral administration of oil from *Allium sativum* bulb was found to be effective in improvement of arsenic-induced keratosis affecting palms and soles of patients (Misbahuddin et al. 2013). Similar complications were also found surpassed by *Nigella sativa* seed oil (Bashar et al. 2014).

**Table 3.** Natural products with clinical arsenic toxicity ameliorative effects.

Sl. No.	Name	Material used (if applicable)	References
1	Vitamin A	–	Ahmad et al., 1998; Khandker et al., 2006
2	Vitamin C	–	Ahmad et al., 1998; Khandker et al., 2006; Melkonian et al., 2012
3	Vitamin E	–	Ahmad et al., 1998; Verret et al., 2005; Khandker et al., 2006; Melkonian et al., 2012
4	*Spirulina*	Whole plant (algae)	Sikder et al., 2000; Khan et al., 2001; Misbahuddin et al., 2006
5	*Allium sativum*	Oil from bulb	Misbahuddin et al., 2013
6	*Nigella sativa*	Oil from seed	Bashar et al., 2014

Improvement of symptoms of arsenicosis patients in Bangladesh have been reported to occur following use of vitamin A, C and E in two studies (Ahmad et al. 1998; Khandker et al. 2006). Vitamin E and selenium either alone or in combination, slightly improved arsenic-induced skin lesions in another study (Verret et al. 2005). Another more recent study in Bangladesh demonstrated vitamin C and E significantly improved arsenic induced keratotic skin lesions in arsenicosis patients (Melkonian et al. 2012). However, no placebo controlled trials with these vitamins have been carried out nor the toxicity of their long-term use has been ascertained.

## Discussion and conclusion

Chronic arsenic toxicity results in multisystem disease. Apart from advising avoiding arsenic contaminated drinking water and certain symptomatic treatments, there are no evidence-based definitive treatment regimens to treat chronic arsenic toxicity in humans. Nevertheless, antioxidants have been advocated (Ratnaike 2003; Mazumder 2008); since the elicitation of oxidative stress by generation of free radicals during the metabolism of arsenic in body is considered to be involved in arsenic toxicity (Shi et al. 2004; Kim et al. 2015).

There is ample literature currently available on usefulness of crude medicinal plant extracts against experimental arsenic and other heavy-metal poisoning. These extracts in general exhibit antioxidant properties and thus show potential in reducing metal/metalloid induced oxidative stress. Most of the literature neither talk about their usefulness or capability in reducing body arsenic burden nor make any attempt to isolate, identify, or characterize the active constituent(s). This is the major shortcoming of most of these studies.

Present literature study revealed that all of the medicinal plants and natural products possessing arsenic toxicity alleviative effects simultaneously demonstrated good intrinsic antioxidant effect by suppression of arsenic-induced oxidative stress by multimodal augmentation of endogenous defence mechanisms that resulted in amelioration from arsenic toxicity. The 14 natural products (phytochemicals) tested are established nutraceuticals and these are all well reported as natural antioxidants. This indicates the beneficial role of antioxidant supplementation and strongly corroborates with the recommendation of antioxidant therapy to humans. However, the benefits of these compounds at cellular level need validation in human subjects with chronic arsenic toxicity.

In groundwater, arsenic may exist inorganically as both trivalent and pentavalent forms. Although both forms are potentially harmful to human health, trivalent arsenic is more toxic (Kapaj et al. 2006). In all the studies the test agents alleviated trivalent arsenical, i.e., sodium arsenite or arsenic trioxide-induced toxicity indicating their possible promise in management of groundwater arsenic toxicity in humans. Although all of these studies are pre-clinical and short term, few of these natural products, namely, vitamin A, C and E have already shown protective effect in clinical studies also.

So far the most studied combination of vitamin C and E in rodents and humans as well require further definitive clinical exploitation. More of such pre-clinically proven natural products should be introduced for clinical studies. These agents could be used alone or together with chelating agents (Flora et al. 2007). These agents may aid in disease reversal or may serve as disease modifying agents and thus could help in reducing the patient’s sufferings.

It is firmly believed that the present facts and findings, though principally observed in animal models, will have sustainable curative potential among the already afflicted populations, neutralizing impact on freshly emerging arsenicosis scenario and possible proactive prevention to those potentially susceptible to arsenicals exposure (Jomova et al. 2011). This research could lead to discovery of any potentially useful agent in clinical management of arsenicosis in humans in due course, which may act by distinct mechanism other than chelation like oxidative stress or apoptosis modulation. The current findings are quite encouraging for further mechanistic preclinical and appropriately designed clinical studies on medicinal plants and natural products especially, in management of chronic arsenic toxicity in humans.
