# Corrigendum

**DOI:** 10.1080/13880209.2017.1284400

**Published:** 2017-01-31

Sanjib Bhattacharya (2017). Medicinal plants and natural products in amelioration of arsenic toxicity: a short review, *Pharmaceutical Biology*.

http://dx.doi.org/10.1080/13880209.2016.1235207

The above article was first published online with the below errors:

The author’s name should be 'Sanjib Bhattacharya' instead of 'Sanijb Bhattacharya'.

